# *Bifidobacterium* Strain-Specific Enhances the Efficacy of Cancer Therapeutics in Tumor-Bearing Mice

**DOI:** 10.3390/cancers13050957

**Published:** 2021-02-25

**Authors:** Youngmin Yoon, Gihyeon Kim, Bu-Nam Jeon, Sungsoon Fang, Hansoo Park

**Affiliations:** 1 Department of Biomedical Science and Engineering, Gwangju Institute of Science and Technology (GIST), Gwangju 61005, Korea; korean8503@gm.gist.ac.kr (Y.Y.); kkra12@gm.gist.ac.kr (G.K.); 2 Genome and Company, Pangyo-ro 255, Bundang-gu, Seoungnam 13486, Korea; junbnx@genomecom.co.kr; 3 Severance Biomedical Science Institute, BK21 PLUS Project for Medical Science, Gangnam Severance Hospital, Yonsei University College of Medicine, Seoul 06273, Korea; sfang@yuhs.ac

**Keywords:** colorectal cancer, *Bifidobacterium breve*, oxaliplatin, PD-1 blockade, transcriptomic analysis

## Abstract

**Simple Summary:**

The efficacy of cancer therapeutics depends on several factors, including the tumor genome, epigenome, and transcriptome. In addition, the tumor microenvironment, which consists mainly of immune cells, can influence cancer treatment outcomes. Hence, effectively leveraging host immunity is an important aspect of cancer treatment strategies. The human gut microbiome is involved in the regulation of the immune responses and affects the efficacy of chemotherapeutic and immunotherapeutic agents, including oxaliplatin, cyclophosphamide, and immune checkpoint inhibitors. This study reveals an additional dimension to the *Bifidobacterium* strain-specific determination of anti-cancer therapeutic efficacy using flow cytometry and transcriptome analysis of bacterial strain-fed mice and bacterial whole transcriptome analysis. We hope that our work will contribute to leveraging the gut microbiome to improve anti-cancer therapies.

**Abstract:**

Colorectal cancer (CRC) is among the leading causes of cancer-related death in the world. The development of CRC is associated with smoking, diet, and microbial exposure. Previous studies have shown that dysbiosis of the gut microbiome affects cancer development, because it leads to inflammation and genotoxicity. Supplementation with specific microbiota induces anti-tumor effects by enhancing of anti-tumor immunity. Here, we observed that supplementation with either of two *B. breve* strains reduces tumor growth in MC38 colon carcinoma-bearing mice. Interestingly, only one *B. breve* strain boosted the efficacy of cancer therapeutics, including oxaliplatin and PD-1 blockade. Extensive immune profiling and transcriptomic analysis revealed that the boosting *B. breve* strain augments lymphocyte-mediated anti-cancer immunity. Our results suggest that supplementation with *B. breve* strains could potentially be used as a strategy to enhance the efficacy of CRC therapeutics.

## 1. Introduction

It is increasingly reported that the gut microbiota plays a critical role in host metabolism and immunity [1], and that the gut microbiome composition changes with age, diet, and diseases [2,3]. Colorectal cancer (CRC) is among the leading causes of cancer death in men and women in the United States of America (USA). The development of CRC is related to genetic and epigenetic alterations, inflammation, and microbial exposure [4,5,6]. Specific gut microbiota, such as *Fusobacterium nucleatum*, enterotoxigenic *Bacteroides fragilis*, and *Escherichia coli* are correlated with the development of intestinal adenomas and carcinomas, because they damage the DNA and affect immune and intestinal barrier functions [7,8,9]. Several studies have demonstrated that supplementation with gut microbiota species, including *Enterococcus hirae*, *Lactobacillus johnsonii*, and *Bifidobacterium* species reinforces the efficacy of platinum-based chemotherapy, cyclophosphamide, and immunotherapy by enhancing anti-tumor immune responses [10,11,12].

*Bifidobacterium* is the dominant member of the early life gut microbiome, mainly consisting of *B. infantis*, *B. longum*, and *B. breve* [13,14,15]. As an important part of the gut microbiome in infants, *Bifidobacterium* spp. reduce the risk of intestinal infections, including neonatal necrotizing enterocolitis, by regulating immune responses and protecting against intestinal barrier dysfunction [16,17,18]. *Bifidobacterium* spp. also affect immunotherapy responses, and supplementation with *Bifidobacterium* spp. in a mouse cancer model significantly improved anti-tumor immunity by CD8^+^ T cells [12]. In this study, we identified *B. breve* strains that influence cancer growth and the efficacy of cancer therapeutics through immune activation, and further investigated the underlying genetic mechanisms. Our results provide important insights regarding the gut microbiome and its applications in cancer patients.

## 2. Materials and Methods

### 2.1. Mice

All animal experiments were performed with approval from the Institutional Animal Care and Use Committee of CHA University (permission number IACIC180010). Mice used in this study were maintained and handled according to policies approved by CHA University. Five-week-old female C57B6/N mice were purchased from the Orient Bio (Gapyeong, Gyeonggi, Korea). For the tumor mice model, mice were orally administered *Bifidobacterium* strains for 14 days (−14 day). Then, mice were implanted with 2 × 10^5^ MC38 colon adenocarcinoma cells subcutaneously and treated with 3 mg/kg oxaliplatin (S1224, Selleckchem, Houston, TX, USA) or 2 mg/kg anti-PD-1 mAb (clone RMP1-14, BioXCell, USA) intraperitoneally. Syngeneic tumor mice were treated 6 times with anti-PD-1 mAb or oxaliplatin on days 3, 7, 10, 14, 17, and 21. Tumor size was monitored three times a week until the study endpoint. Tumor volume was calculated as length × width^2^ × 0.5.

### 2.2. Cell Culture

MC38 cells were purchased from Kerafast (Cat# EZH204, Winston-Salem, NC, USA) and authenticated using DNA fingerprint analysis. All cells were free of mycoplasma contamination. Cells were cultured in Dulbecco’s Modified Eagle Medium (DMEM, GIBCO) with 10% fetal bovine serum (FBS, GIBCO) and 100 units/mL penicillin (GIBCO) and streptomycin (GIBCO). All cells were maintained in a humidified incubator at 37 °C in a 5% CO_2_ atmosphere.

### 2.3. Bacteria

*B. breve* JCM92 (*B. bre* JCM92) were purchased from the Japan Culture Collection of Microorganisms (JCM). *B. breve* Bb03 (*B. bre* Bb03) were purchased from DuPont (Wilmington, NC USA). To verify bacterial viability, we cultured aliquoted cells on BL (Blood Liver) agar plates at 37 °C in anaerobic conditions (O_2_ < 1 ppm, 5% CO_2_, 5% N_2_) for 48 h and counted viable colonies (viable cells). For in vivo experiments, each *B. breve* strain was cultured in BL medium (Kisanbio, Seoul, Korea) at 37 °C in an anaerobic condition (O_2_ < 1 ppm, 5% CO_2_, 5% N_2_) for 48 h. After culture, the bacteria were lyophilized for preservation. Next, we measured colony forming unit (CFU) counts using a PBS serial dilution method (CORNING, 21-040-CVR). For each sampling point, an aliquot of culture was diluted to 10^−6^, 10^−7^, and 10^−8^, and 100 μL was spread on BL agar plates. After counting CFUs, each *B. breve* strain was divided into daily administration doses and stored at 4 °C. Each *B. breve* strain was treated at 1 × 10^9^ CFU orally once daily. For bacterial whole transcriptome analysis, each *B. breve* strain was harvested in the exponential phase. Then, we cultured two *B. breve* strains in BL agar media at 37 °C in anaerobic conditions (O_2_ < 1 ppm, 5% CO_2_, 5% N_2_) for 48 h. After colony isolation from the BL agar plate, the colony was cultured in BL broth media at 37 °C for 48 h under anaerobic conditions (O_2_ < 1 ppm, 5% CO_2_, 5% N_2_).

### 2.4. Flow Cytometry Analysis

Tissues from experimental mice were obtained at day 15 after tumor cell implantation. Tumors were dissected into small pieces and transferred into RPMI 1640 media (GIBCO) supplemented with 2.5 mg/mL collagenase I, 1.5 mg/mL collagenase II, 1 mg/mL collagenase IV, 50 μg/mL DNase 1, and 0.25 mg/mL hyaluronidase Type IV-S. The tissues were incubated at 37 °C for 50 min and filtered using a 70 μm cell strainer (BD Bioscience, San Jose, CA, USA). Spleens were homogenized in RPMI 1640 media, incubated in red blood cell (RBC) lysis buffer (eBioscience, San Diego, CA, USA) and filtered using a 70 μm cell strainer. Splenocytes and tumor cells were incubated with anti-mouse CD16/CD32 (BD Bioscience) for 10 min at 4 °C to block the Fc receptor. After evaluating cell viability and surface staining, fixation/permeabilization buffer solution (BioLegend, San Diego, CA) was added. Splenocytes and tumor cells were stained with the following mouse antibodies: anti-CD45 (Biolegend, Cat# 103116), CD3 (Biolegend, Cat# 100218), NK1.1 (Biolegend, Cat# 108708), CD49b (Biolegend, Cat# 108910), CD4 (Biolegend, Cat#100422), CD25 (Biolegend, Cat#101904), Foxp3 (Invitrogen, Waltham, MA, Cat#17-5773-82), CD44 (Biolegend, Cat# 103008), CD62L (Biolegend, Cat#104412), and CD8a (Biolegend, Cat#100706). Cell acquisition was performed on a CANTO II flow cytometer (BD Bioscience, Franklin Lakes, NJ, USA) and data were analyzed using FlowJo software (TreeStar, San Carlos, CA, USA).

### 2.5. Quantitative PCR (qPCR) Analysis of Tumor Tissue

Total RNA from mice tumors was extracted using an RNeasy Plus Mini Kit (Qiagen, Germantown, MD) and reverse transcription was conducted using a PrimeScript First Strand cDNA Synthesis Kit (Takara, Mountain View, CA). qPCR analysis was performed using SYBR Premix Ex Taq (Tli RNase H Plus) (Takara) and a CFX384 Touch instrument (Bio-Rad, Hercules, CA, USA). The primers used in this study: IFN-γ, 5′- GAAAGCCTAGAAAGTCTGAATAACT-3′ and 5′- ATCAGCAGCGACTCCTTTTCCGCTT-3′; IL-2, 5′-ATGTACAGCATGCAGCTCGCATC-3′ and 5′-GGCTTGTTGAGATGATGCTTTGACA-3′; IL-10, 5′-TGAAGACCCTCAGGATGCGG-3′ and 5′-AGAGCTCTGTCTAGGTCCTGG-3′; and β-actin, 5′- CGTGCGTGACATCAAAGAGAA-3′ and 5′-TGGATGCCACAGGATTCCAT-3′. PCR conditions were as follows. For IFN-γ, IL-2, and β-actin: initial denaturation at 95 °C for 1 min; 35 cycles of denaturation (95 °C for 30 s), annealing (60 °C for 30 s), and extension (72 °C for 1 min), and a final extension at 72 °C for 1 min. For IL-10: initial denaturation at 95 °C for 1 min, 40 cycles of denaturation (95 °C for 30 s), annealing (60 °C for 30 s), and extension (72 °C for 1 min); and a final extension at 72 °C for 1 min.

### 2.6. Mice RNA Sequencing and Data Analysis

Intestines were harvested at day 22 after MC38 tumor cell inoculation, and an RNeasy Mini Kit (Qiagen) was used to extract RNA. After RNA extraction, 151-bp paired-end libraries were constructed from 1 μg RNA using a TruSeq RNA Sample Prep Kit v2 (Illumina, San Diego, CA). Whole-RNA sequencing was performed on an Illumina HiSeq instrument. RNA-seq reads were aligned to the mouse reference genome (GRCm38) using the STAR aligner [19]. Quantification of gene expression and differential expression analysis were performed using RSEM [20] and the edgeR package [21]. The ClueGO plug-in (v2.5.4, http://www.ici.upmc.fr/cluego/, 1 January 2009) in Cytoscape software (v3.3.0, http://cytoscape.org/, 1 January 2003) was used to analyze gene ontology (GO) [22]. Functionally-related GO terms for biological processes in *Mus musculus* (version: 27 February 2019) were grouped based on a kappa score > 0.4 and a network specificity of 5–10 using GO term fusion. Statistical significance was calculated using a two-sided hypergeometric test, and the false discovery rate was corrected using the Bonferroni step-down method.

### 2.7. Bacterial RNA Sequencing and Data Analysis

Bacterial RNA was extracted using the ZymoBIOMICS RNA Miniprep Kit (Zymo Research, Irvine, CA). Sequencing and library construction were performed on the Illumina Hiseq 2500 with 101 bp paired-end. Ribosomal RNA was removed using the Ribo-Zero™ rRNA Removal Kit (Bacteria) (Epicentre, Madison, WI). Libraries were prepared with the TruSeq RNA Sample Prep kit v2 (Illumina). RNA-sequenced reads were mapped on the reference genome of *Bifidobacterium breve* (NZ_AP012324.1) using STAR with alignIntron MAX 1 [19]. Then, the mapped reads were used to calculate read counts of genes using cufflinks [23], and the gene list was inputted into Cytoscape plug-in ClueGO v2.5.4 [22] to annotate functionally grouped networks. Functionally related GO terms for biological processes in *Escherichia coli* (version: November 18, 2016) were grouped based on a kappa score greater than 0.4 with a network specificity of 4–10. Statistical significance was calculated using two-sided hypergeometric tests, and the false discovery rate was corrected using the Bonferroni step down method.

### 2.8. Statistical Analysis

Statistical analyses were performed using GraphPad Prism 8.4.3. Differences between groups were analyzed using one-way or two-way ANOVA with Tukey’s post-test. Differences between groups were considered significant if *p* < 0.05. Statistical details are provided in the figure legends.

## 3. Results

### 3.1. Mice Supplemented with B. breve Strains Exhibit Anti-Tumor Effects

**Figure 1:**
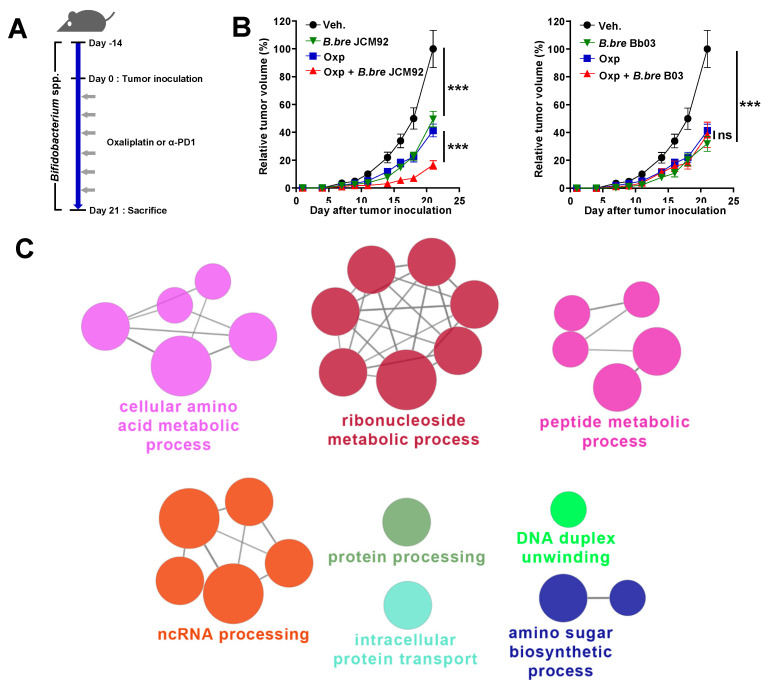
Supplementation of *Bifidobacterium* strains exhibits anti-tumor effects. (**A**) Experimental protocol: each *Bifidobacterium* strain was administered orally 14 days before MC38 inoculation and continued until the end of the experiment. After tumor inoculation, oxaliplatin or PD-1 blockade was injected intraperitoneally. (**B**) Tumor growth curves after administering *Bifidobacterium* strains, with or without oxaliplatin. Oxp, oxaliplatin; *B. bre* JCM92, *B. breve* JCM92; *B. bre* Bb03, *B. breve* Bb03. Data are expressed as the means ± standard error of means (SEM). *p*-values were determined by two-way ANOVA using Tukey’s post-test. (**C**) Representation of the ClueGO functional network analysis of the upregulated genes in *B. bre* JCM92 compared to *B. bre* Bb03 (Fold change > 2). *** *p* < 0.001, and ns, not significant.

To examine the anti-tumor effects of *Bifidobacterium* spp. on colon cancer, we administered *Bifidobacterium* strains alone (*B. breve* JCM1192, *B. bre* JCM92; *B. breve* Bb03, *B. bre* Bb03) with oxaliplatin to MC38 colon carcinoma-bearing mice (Figure 1A and Table S1). Oral administration of *B. breve* in tumor-bearing mice significantly decreased tumor growth, compared to mice treated with the vehicle (phosphate-buffered saline; PBS) (Figure 1B). To determine the relationship between *B. breve* strain supplementation and cancer therapeutics, we treated colon carcinoma-bearing mice with each *B. breve* strain alone and in combination with oxaliplatin (Figure 1A). Oxaliplatin is a third-generation platinum-derivative chemotherapeutic agent and is an important metastatic CRC medication [24,25]. Interestingly, when mice were treated with oxaliplatin and *B. breve* strains, only mice treated with *B. bre* JCM92 showed significantly decreased tumor growth compared to mice treated with oxaliplatin alone (Figure 1B). To understand why only *B. bre* JCM92 enhanced the effect of oxaliplatin, we performed RNA sequencing of *B. bre* JCM92 (boosting strain) and *B. bre* Bb03 (non-boosting strain). We observed that 88 genes were upregulated in *B. bre* JCM92 compared to *B. bre* Bb03 (Table S2, Fold Change > 2). ClueGO analysis showed that 27 terms, including protein processing, intracellular protein transport, cellular amino acid metabolic process, ribonucleoside metabolic process, and peptide metabolic process were significantly enriched in *B. bre* JCM92 (Figure 1C and Table S3).

### 3.2. B. bre JCM92 Boosts the Efficacy of Oxaliplatin by Enhancing Anti-Tumor Immunity

**Figure 2:**
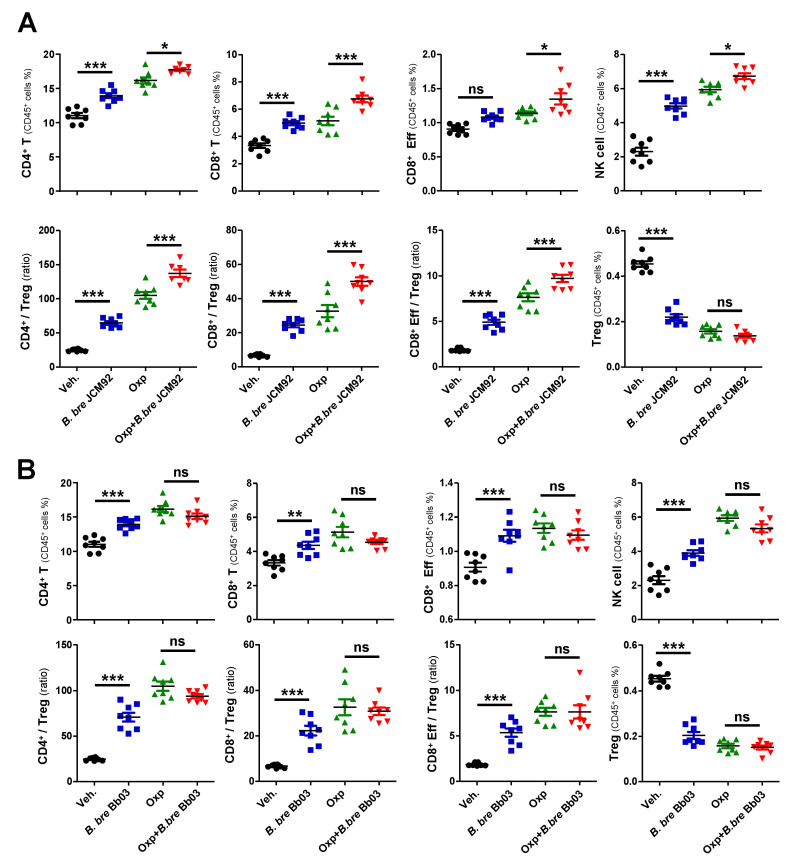
*B. bre* JCM92 boosts oxaliplatin efficacy by enhancing anti-tumor immunity in the spleen. (**A**,**B**) Immune cell profiling of the spleen in mice treated with oxaliplatin in combination with *B. bre* JCM92 (**A**) or *B. bre* Bb03 (**B**) using flow cytometry analysis. Data are expressed as the means ± SEM. *p*-values were determined by one-way ANOVA using Tukey’s post-test. * *p* < 0.05, ** *p* < 0.001, *** *p* < 0.001, and ns, not significant.

To investigate the mechanism by which *Bifidobacterium* strains induce anti-tumor effects, we performed immune cell profiling of spleen and tumor tissue 15 days after tumor transplantation. Mice treated with *B. bre* JCM92 and *B. bre* Bb03 had increased CD4^+^ T, CD8^+^ T, and effector CD8^+^ T, and NK cells. These mice also had an increased ratio of CD4^+^/regulatory T cells (Tregs), CD8^+^/Tregs, and effector CD8^+^/Tregs. However, the mice had decreased Treg counts in splenocytes (Figure 2A,B). When we compared immune cell profiling between oxaliplatin alone and oxaliplatin + *B. breve*-treated groups, *B. bre* JCM92 enhanced the effect of oxaliplatin by increasing CD4^+^ T, CD8^+^ T, NK cells and the CD4^+^/Treg, CD8^+^/Treg, and effector CD8^+^/Treg ratios (Figure 2A). However, *B. bre* Bb03 did not boost the anti-tumor effect of oxaliplatin (Figure 2B).

**Figure 3:**
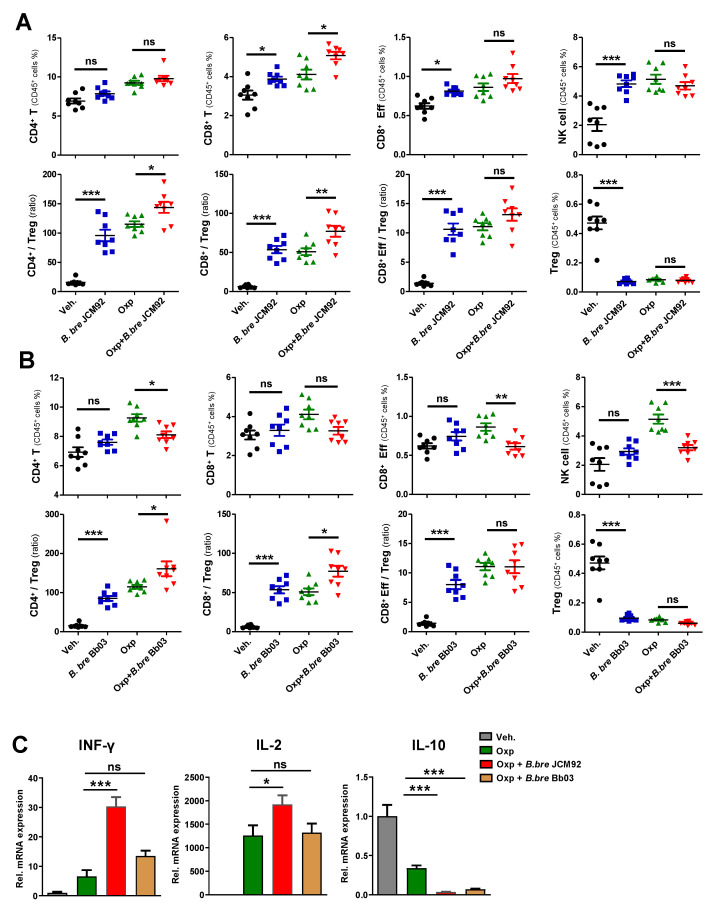
*B. bre* JCM92 boosts oxaliplatin efficacy by enhancing anti-tumor immunity. (**A**,**B**) Immune cell profiling of tumor tissue in mice treated with oxaliplatin in combination with *B. bre* JCM92 (**A**) or *B. bre* Bb03 (**B**) using flow cytometry analysis. Data are expressed as the means ± SEM. *p*-values were determined by one-way ANOVA using Tukey’s post-test. (**C**) Expression of intratumoral cytokines in mice treated with oxaliplatin combined with *B. bre* JCM92 or *B. bre* Bb03 were measured using qPCR. Data are expressed as the means ± SEM. *p*-values were determined by one-way ANOVA using Tukey’s post-test within oxaliplatin treated groups. For all graphs, * *p* < 0.05, ** *p* < 0.01, *** *p* < 0.001, and ns, not significant.

Immune cell profiling of tumor-infiltrating lymphocytes (TILs) revealed that *B. bre* JCM92 and *B. bre* Bb03 significantly increased the CD4^+^/Treg, CD8^+^/Treg, and the effector CD8^+^/Treg ratio (Figure 3A,B). Furthermore, oxaliplatin combined with *B. bre* JCM92 significantly increased CD8^+^ T cells and the CD4^+^/Treg and CD8^+^/Treg ratios (Figure 3A,B). Next, we examined intra-tumor cytokine expression using qPCR. Interferon-gamma (IFN-γ) and interleukin-2 (IL-2) expression in tumor tissue was much higher in mice treated with oxaliplatin with or without *B. breve* strains (*B. bre* JCM92 and *B. bre* Bb03) compared to IFN-γ and IL-2 expression in vehicle-treated mice. Notably, mice treated with combined oxaliplatin and *B. bre* JCM92 exhibited significantly higher IFN-γ and IL-2 expression than mice treated with oxaliplatin alone (Figure 3C). In contrast, interleukin 10 (IL-10) expression was much lower in the mice treated with oxaliplatin with or without *B. breve* strains than in mice treated with the vehicle (Figure 3C). These results demonstrate that *B. breve* monotherapy induces anti-tumor effects by augmenting anti-tumor immunity, but only *B. bre* JCM92 enhances the anti-tumor effects of oxaliplatin.

### 3.3. B. bre JCM92 Boosts the Efficacy of PD-1 Blockade by Enhancing Anti-Tumor Immunity

**Figure 4:**
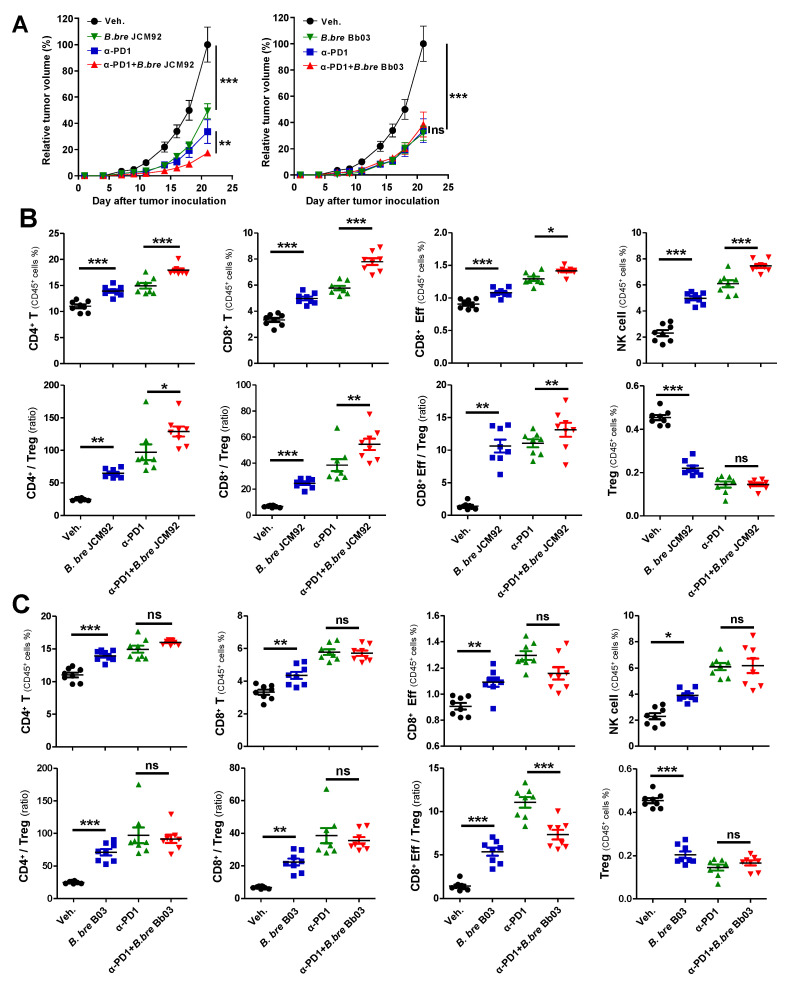
*B. bre* JCM92 boosted PD-1 blockade efficacy. (**A**) Tumor growth curves after administering *B. breve* strains, with or without PD-1 blockade. Data are expressed as the means ± SEM. *p*-values were determined by two-way ANOVA using Tukey’s post-test. (**B**,**C**) Immune cell profiling of spleen tissue in mice treated with PD-1 blockade in combination with *B. bre* JCM92 (**B**) or *B. bre* Bb03 (**C**) using flow cytometry analysis. Data are expressed as the means ± SEM. *p*-values were determined by one-way ANOVA using Tukey’s post-test. For all graphs, * *p* < 0.05, ** *p* < 0.01, *** *p* < 0.001, and ns, not significant.

Several studies have revealed that the gut microbiota enhances the response of immune checkpoint inhibitors that target cytotoxic T lymphocyte antigen-4 (CTLA-4) and programmed cell death protein-1 (PD-1) [12,26]. Therefore, we tested whether *Bifidobacterium* strains boost the efficacy of PD-1 blockade (Figure 4A). Similar to previous results, mice treated with a PD-1 blockade and *B. bre* JCM92 exhibited significantly decreased tumor growth compared to mice treated with PD-1 blockade alone (Figure 4A).

**Figure 5:**
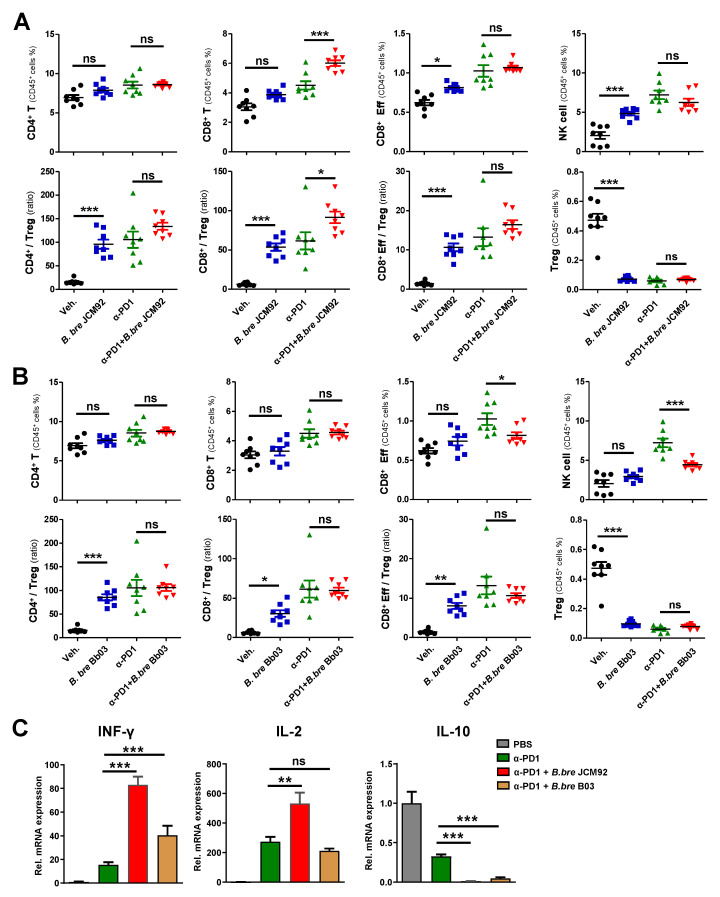
*B. bre* JCM92 boosts PD-1 blockade efficacy by enhancing anti-tumor immunity in tumor tissue. (**A**,**B**) Immune cell profiling of tumor in mice treated with PD-1 blockade in combination with *B. bre* JCM92 (**A**) or *B. bre* Bb03 (**B**) using flow cytometry analysis. (**C**) Expression of intra-tumoral cytokines in mice treated with PD-1 blockade in combination with *B. bre* JCM92 or *B. bre* Bb03 were measured using qPCR. Data are expressed as the means ± SEM. *p*-values were determined by one-way ANOVA using Tukey’s post-test within the PD-1 blockade-treated groups. * *p* < 0.05, ** *p* < 0.01, *** *p* < 0.001, and ns, not significant.

Splenic immune cell profiling showed that combined PD-1 blockade and *B. bre* JCM92 significantly enhanced anti-tumor immunity by increasing CD8^+^ T, effector CD8^+^ T, and NK cells. Additionally, combined PD-1 blockade and *B. bre* JCM92 increased the CD8^+^/Treg and effector CD8^+^/Treg ratios compared to PD-1 blockade alone (Figure 4B). In tumor tissue, PD-1 blockade combined with *B. bre* JCM92 significantly increased the CD8^+^ T cell level and the CD8^+^/Treg ratio compared to PD-1 blockade alone (Figure 5A). However, immune cell profiling of the spleen and tumor tissue revealed that *B. bre* Bb03 did not enhance the anti-tumor immunity of PD-1 blockade (Figure 4C and Figure 5B). Intra-tumoral cytokine expression revealed that mice treated with PD-1 blockade and *B. bre* JCM92 had much higher IFN-γ and IL-2 expression compared to mice treated with PD-1 blockade alone (Figure 5C). Although IFN-γ expression was significantly enhanced in mice treated with PD-1 blockade combined with *B. bre* Bb03, the combination of PD-1 blockade and *B. bre* JCM92 resulted in higher IFN-γ expression (Figure 5C). IL-10 expression was significantly decreased in mice treated with PD-1 blockade and either *B. breve* strain (Figure 5C).

### 3.4. Transcriptome Analysis of Intestinal Tissue Reveals That B. bre JCM92 Has the Ability to Boost the Effects of Cancer Therapeutics

**Figure 6:**
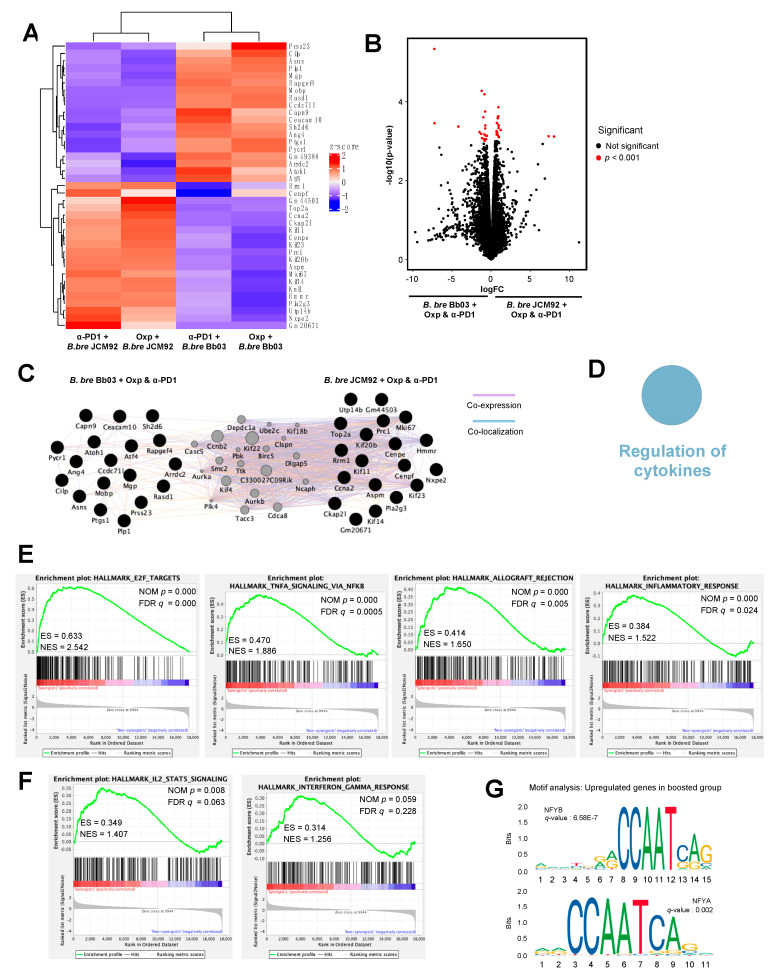
Effect of cancer therapeutics and *B. breve* strains on the intestinal transcriptome. (**A**) Heat map showing hierarchical clustering of differentially expressed genes (DEGs) between mice treated with cancer therapeutics (oxaliplatin or PD-1 blockade) and *B. bre* JCM92 or *B. bre* Bb03. (**B**) Volcano plots illustrating genes between mice treated with cancer therapeutics and *B. bre* JCM92 and mice treated with cancer therapeutics and *B. bre* Bb03. Red dots represent significantly different genes (*p* < 0.001). (**C**) Network analysis of DEGs from mice treated with cancer therapeutics and *B. bre* JCM92, and mice treated with cancer therapeutics and *B. bre* Bb03 using Cytoscape. Co-expressed and co-localized genes are indicated by purple and blue lines, respectively. (**D**) Representation of the ClueGO functional network analysis shows upregulated genes in mice treated with cancer therapeutics and *B. bre* JCM92 compared to mice treated with cancer therapeutics and *B. bre* Bb03. (**E**,**F**) GSEA of intestinal transcriptome data exhibited that mice treated with cancer therapeutics and *B. bre* JCM92 were significantly enriched in hallmark pathways compared to mice treated with cancer therapeutics and *B. bre* Bb03. (**G**) Motifs within upregulated genes of mice treated with cancer therapeutics and *B. bre* JCM92. NFYB, nuclear transcription factor Y subunit beta; NFYA, nuclear transcription factor Y subunit alpha.

To understand the mechanism by which the administration of the gut microbiome influences the intestine and anti-tumor immune responses, we performed whole transcriptome sequencing on the intestine of mice treated with cancer therapeutics and *B. bre* JCM92 and *B. bre* Bb03. Hierarchical clustering analysis revealed that intestinal gene-expression patterns were distinct between mice treated with *B. bre* JCM92 and *B. bre* Bb03 (Figure 6A). We analyzed differentially expressed genes (DEGs) between the enhanced (oxaliplatin + *B. bre* JCM92 and PD-1 blockade + *B. bre* JCM92) and unenhanced groups (oxaliplatin + *B. bre* Bb03 and PD-1 blockade + *B. bre* Bb03; *p* < 0.001; Figure 6B). Cytoscape network visualization exhibited that DEGs from mice treated with cancer therapeutics and *B. breve* strains (*B. bre* JCM92 and *B. bre* Bb03) are biologically related (Figure 6C). ClueGO analysis revealed that the term “Regulation of cytokines” was significantly enriched in mice treated with cancer therapeutics combined with *B. bre* JCM92 compared to mice treated with cancer therapeutics and *B. bre* Bb03 (Figure 6D and Table S4). Next, we performed gene set enrichment analysis (GSEA) between the boosted group and non-boosted group. The significantly enriched gene signatures in mice treated with the cancer therapeutics (oxaliplatin and PD-1 blockade) and *B. bre* JCM92 compared to mice treated with cancer therapeutics and *B. bre* Bb03 included gene sets involving E2F targets, tumor necrosis factor-α (TNF-α) signaling via nuclear factor kappa B (NFκB), allograft rejection, and inflammatory response (Figure 6E and Figure S1). Consistent with tumor cytokine expression, mice treated with cancer therapeutics and *B. bre* JCM92 exhibited higher IL-2, STAT5 signaling, and IFN-γ response compared to mice treated with oxaliplatin or PD-1 blockade and *B. bre* Bb03 (Figure 6F and Figure S2). Transcription factors regulate transcription initiation by recognizing specific binding sites, and transcription factor dysregulation is associated with human cancers [27,28,29]. To understand the regulatory mechanism of the gut microbiome, motif analysis of the intestinal transcriptome was performed. Nuclear transcription factor Y subunit beta and nuclear transcription factor Y subunit alpha genes were significantly associated with the upregulated genes in mice treated with the combination of cancer therapeutics and *B. bre* JCM92 (boosted group; Figure 6G). Nuclear factor Y binds to the interleukin-4 (IL-4) promoter and regulates IL-4 gene expression [30,31]. IL-4 regulates lymphocyte expansion and function [32] and exhibits anti-tumor activity by promoting Gr-1^+^ granulocyte and CD8^+^ T cell maturation [33]. These results reveal that supplementation of *B. breve* affects intestinal gene expression and that the changes are associated with anti-tumor immune responses.

## 4. Discussion

The gut microbiome affects immune system development and differentiation by regulating innate immune responses, including innate lymphoid cells and gene expression [34]. Several diseases, such as cancer, obesity, and inflammatory bowel diseases are correlated with intestinal dysbiosis [34,35]. Previous studies suggest that supplementation with probiotics influences host homeostasis and has a therapeutic effect on immune-mediated diseases [36,37]. *Bifidobacterium* spp. are among the most frequently used as probiotics and have favorable effects in several diseases, including ulcerative colitis and enteropathogenic *E. coli* O157 infection [38,39].

Here, we found that *Bifidobacterium* strains confer anti-tumor properties. Although two *B. breve* strains increased anti-tumor immunity, only one *B. breve* strain boosted cancer therapeutic efficacy. Immune cell profiling of spleen and tumor tissue revealed that *B. breve* strains alone augmented anti-tumor immunity by increasing CD8^+^ T and effector CD8^+^ T cell numbers, and by increasing the CD8^+^/Treg and effector CD8^+^/Treg ratio. However, only *B. bre* JCM92 boosted the efficacy of cancer therapeutics.

Interestingly, immune cell profiling exhibited that spleens from mice treated with *B. bre* JCM92 and PD-1 blockade had significantly increased CD4^+^ and CD8^+^ T cells compared to PD-1 monotherapy (Figure 4B), whereas tumors from mice treated with *B. bre* JCM92 and PD-1 blockade only showed enhanced CD8^+^ T cells compared to PD-1 monotherapy (Figure 5A). We hypothesize that the CD4^+^ T cells activate CD8^+^ cells rather than directly killing tumor cells. Most CD4^+^ T cells could not recognize cancer cells directly due to a lack of MHCII (Major Histocompatibility complex II) in most solid cancer cells (except melanoma and breast cancer [40]), whereas CD4^+^ and CD8^+^ T cells are activated in spleens under immune activation and inflammatory conditions [41]. For those reasons, increased CD4^+^ and CD8^+^ T were observed from splenocytes analysis, while only CD8^+^ T cells were increased in the tumor environment.

Using bacterial transcriptome analysis, we observed that the boosting *B. bre* JCM92 expressed genes were significantly enriched in the ribonucleoside metabolic process, cellular amino acid metabolic process, and amino sugar biosynthetic process pathways compared to non-boosting *B. bre* Bb03. Previous studies showed that amino acid degrading enzymes have anti-tumor effects in preclinical and clinical trials [36,37]. The difference in gene expression between the two *B. breve* strains may be related to the boosting effects of *B. bre* JCM92. However, the exact anti-cancer molecular mechanisms and metabolic processes in *B. bre* JCM92 need to be further investigated. When bacteria are orally administered, the intestine is the primary colonization site [42,43]. Previous studies also showed that the gut microbiome composition is an important factor in determining the response to cancer therapeutics, especially immunotherapy. For instance, *Bifidobacterium* spp., *Ruminococcaceae* and *Faecalibacterium* are significantly enriched in melanoma patients who respond to anti-PD-1 treatment [44,45]. In addition, *Akkermansia muciniphila* abundance is associated with anti-PD-1 treatment response in epithelial tumors [46]. Therefore, we hypothesize that the boosting effects of *B. breve* appear by altering intestinal gene expression. Intestinal transcriptome analysis showed that mice treated with cancer therapeutics and *B. bre* JCM92 showed significantly enriched E2F targets, TNF-α signaling via NFκB, inflammatory responses, and IFN-γ responses. The E2F family of transcription factors plays an important role as transcriptional regulators of cell cycle-dependent gene expression [47], and also modulate apoptosis, metabolism, and angiogenesis in cancer [48,49]. E2F expression in cancer shows conflicting results in both tumor suppression and progression [50,51]. However, the relationship between E2F expression in the intestine and tumors has not yet been investigated. TNF-α is produced by various cells, including macrophages, NK cells, and T cells, and is released in response to peptidoglycan, lipopolysaccharide, and bacterial components [52,53]. Furthermore, TNF-α is involved in apoptosis, cell survival, and inflammation, and is a potential target for cancer therapy [52,54]. Previous studies have shown that TNF-α enhances antitumor effects when used in combination with other cancer therapeutics and cytokines, including adriamycin, actinomycin D, and IFN-γ [55,56]. Similarly to the intestinal transcriptome analysis, intra-tumoral IFN-γ expression was significantly enhanced in mice treated with *B. bre* JCM92 (boosting) and cancer therapeutics compared to mice treated with *B. bre* Bb03 (non-boosting) and cancer therapeutics. IFN-γ is a major player in anti-tumor immunity, and exerts anti-proliferative and pro-apoptotic effects in tumor cells [57,58,59,60], recruits immune cells into the tumor microenvironment [61,62], and enhances the tumoricidal activities of innate and adaptive immune cells [63,64,65]. This cytokine also exhibits anti-angiogenic activity via its anti-proliferative and apoptotic effects on endothelial cells [66,67]. Mutations in IFN-γ signaling genes were identified in tumors resistant to cancer immunotherapy, highlighting the essential role for this protein in chemotherapy responses [68,69]. These results demonstrate that the administration of *B. bre* JCM92 increases anti-tumor immunity by modulating cytokines, including TNF-α and IFN-γ, through the gut–systemic–tumor pathway. However, IL-10 expression was different between intestine and tumors. This difference demonstrates that the anti-tumor effects of *B. breve* are not same in all tissues. We hypothesize that other molecules, including metabolites, underlie these differences. Investigating the underlying mechanisms distinguishing intestine and tumor tissue after *B. breve* treatment needs to be further explored.

## 5. Conclusions

In summary, we identified two *B. breve* strains that show anti-tumor effects by enhancing anti-tumor immunity. Notably, only specific *Bifidobacterium* strains boosted the efficacy of cancer therapeutics. Further study is needed to examine whether supplementation with *Bifidobacterium* strains can be used as supportive anti-tumor drugs to boost the efficacy of cancer therapeutics in clinical trials.

## Data Availability

The data that support the findings of this study are available from the corresponding author, H.P., upon reasonable request.

## Supplementary Materials

The following are available online at https://www.mdpi.com/2072-6694/13/5/957/s1, Figure S1: GSEA of intestine RNA sequencing data; Figure S2: GSEA of intestine RNA sequencing data; Table S1: *Bifidobacterium* strains used in this study; Table S2: Upregulated genes in *B. bre* JCM92 compared to *B. bre* Bb03; Table S3: Gene ontology of DEGs in *B*. *bre* JCM92 compared to *B. bre* Bb03; Table S4: Gene ontology of DEGs in mice treated with cancer therapeutics and *B. bre* JCM92 compared to mice treated with cancer therapeutics and *B. bre* Bb03.
