# Supplementary material for: Bifidobacterium Strain-Specific Enhances the Efficacy of Cancer Therapeutics in Tumor-Bearing Mice
Source: Cancers (Basel). 2021 Feb 25;13(5):957. doi: 10.3390/cancers13050957 (PMC7956760; doi:10.3390/cancers13050957)
Supplement: Supplementary file 1 [file cancers-13-00957-s001.zip › cancers-1098313-supplementary figures.pdf]

cancers

Supplementary Materials: Bifidobacterium Strain-Specific Enhances the Efficacy of Cancer Therapeutics in Tumor-Bearing Mice

Youngmin Yoon, Gihyeon Kim, Bu-Nam Jeon, Sungsoon Fang and Hansoo Park

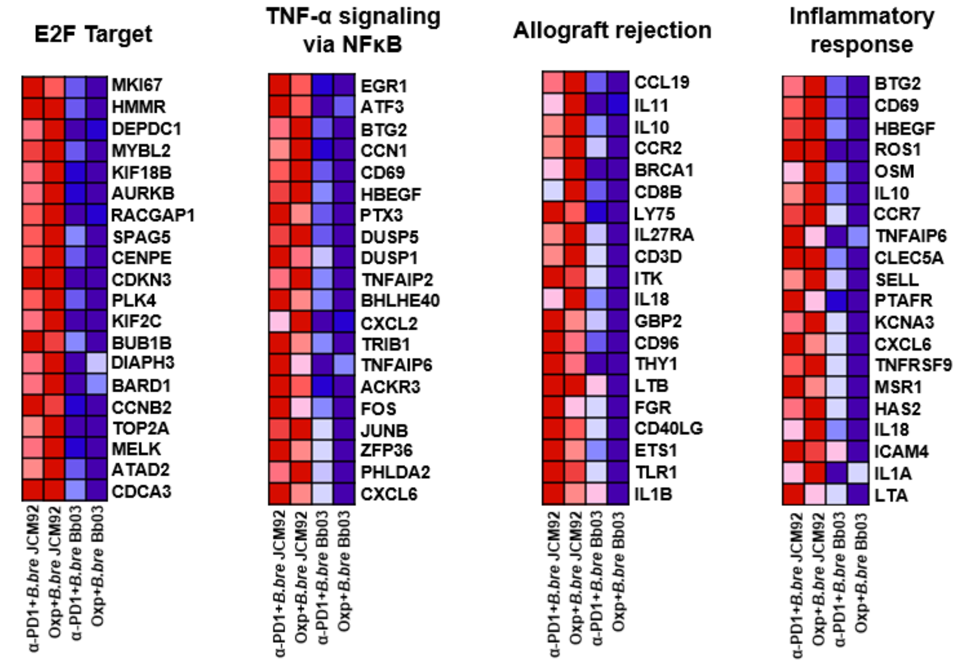

Figure S1. GSEA of intestine RNA sequencing data. The heatmap shows gene expression levels of gene set of E2F targets, TNFα signaling via NFκB, allogenic rejection, and inflammatory response. Red and blue indicate expression levels above and below the median of each gene expression across the samples, respectively.

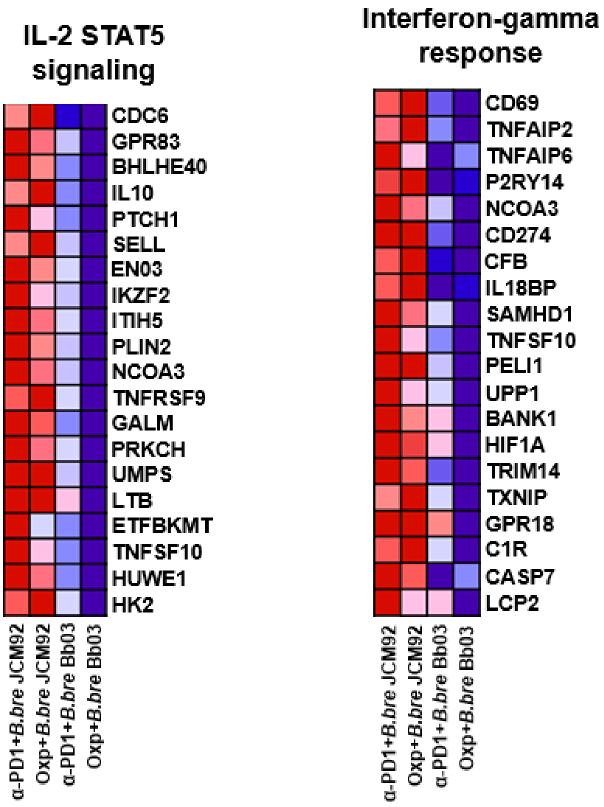

Figure S2. GSEA of intestine RNA sequencing data. The heatmap shows gene expression levels of gene set of IL-2 STAT5 signaling and interferon-gamma response.
